# Preparation and Enzymatic Degradation of Porous Crosslinked Polylactides of Biomass Origin

**DOI:** 10.3390/ijms15069793

**Published:** 2014-06-02

**Authors:** Yuya Kido, Reika Sakai, Baiju John, Masami Okamoto, Jukka V. Seppälä

**Affiliations:** 1 Advanced Polymeric Nanostructured Materials Engineering, Graduate School of Engineering, Toyota Technological Institute, 2-12-1 Hisakata, Tempaku, Nagoya 468-8511, Japan; E-Mails: k.i.d-41-fw@ezweb.ne.jp (Y.K.); perfect_blue0225@hotmail.com (R.S.); jonsbaiju@gmail.com (B.J.); 2 Department of Biotechnology and Chemical Technology, Aalto University, P.O. BOX 16100, Aalto FI-00076, Finland; E-Mail: jukka.seppala@aalto.fi

**Keywords:** crosslinked polylactide, thermoplastic polylactide, pore structure, enzymatic degradation

## Abstract

To understand the enzymatic degradation behavior of crosslinked polylactide (PLA), the preparation and enzymatic degradation of both thermoplastic (linear) and crosslinked PLAs that have pore structures with different dimensions were carried out. The porous structures of the linear PLA samples were of micro and nanoporous nature, and prepared by batch foaming with supercritical CO_2_ and compared with the porous structures of crosslinked PLA (Lait-X) created by the salt leaching method. The surface and cross-sectional morphologies of the porous structures were investigated by using scanning electron microscopy. The morphological analysis of porous Lait-X showed a rapid loss of physical features within 120 h of exposure to proteinase-K enzymatic degradation at 37 °C. Due to the higher affinity for water, enhanced enzymatic activity as compared to the linear PLA porous structures in the micro and nanoporous range was observed.

## 1. Introduction

In recent years, the reduction of carbon dioxide and the extrication from dependence on oil are recognized as the most important issues to reduce the greenhouse effect. Therefore, there is an urgent need to develop plastics from plant origin or plastics that are biodegradable. Among the natural polyesters, polylactides (PLA) have been investigated for drug delivery systems (DDS) in medical applications, degradable sutures, and implants for bone fixation, because of their biocompatibility, easy cell adhesion, and low toxicity. Currently, there is increasing interest in using PLAs for disposable degradable plastic products, drinking bottles, films for agriculture, and so on [1]. However, while they remain very much a niche product at the moment, there are signs that PLAs are ready to be introduced to mass markets via a number of major suppliers, such as Nature Works LLC.

In recent publications, their preparation, characterization, mechanical and various other materials properties [2], biodegradability [3], melt rheology [4], crystallization behavior [5], and finally foam processing [6] of a series of PLA and PLA-based nanocomposites has been reported. Thus, PLA and its nanocomposites hold great promise for future potential applications as high-performance biodegradable materials, and open a new dimension for plastics and composites [7].

As described above, thermoplastic (linear) PLA is becoming increasingly popular. Nonetheless, research on thermoset-based PLA is still at its infancy. Seppälä *et al.* [8] produced sustainable and eco-friendly thermosetting PLA resins. These are easy processable thermoset oligomers that are made from lactic acid and polyol monomers as co-initiator of the crosslinking reaction, and can readily be polymerized to PLA by direct polycondensation with free radicals to produce mechanically excellent polymers. The crosslinked polymers offer a different set of properties from thermoplastics. Often they are hard and rigid, and their production does not necessarily require high temperatures. These materials are very stable because networks are significantly less vulnerable in contrast to their linear counterparts [9].

Apart from this, PLAs with highly porous and interconnected three-dimensional (3-D) structures can potentially be applied as scaffolds for tissue engineering [10]. Several methods have been reported for preparing porous biodegradable polymeric materials by removal of one component from binary phase-separated biodegradable polyester blends via water-extraction of PLA/poly(ethylene oxide) blends [11] or the selective enzymatic degradation of PLA/poly(ε-caprolactone) blends [12]. In a recent publication, Wang *et al.* [13] reported that the fabrication of porous sphere structures, based on crystalline PLA produced via enzymatic degradation of PLA-block-poly(2-ethyl-2-oxazoline) (PEOz)-block-PLA triblock copolymer, where the PEOz-block is a water-soluble polyelectrolyte, is feasible by using the enzyme proteinase-K. The enzymatic degradation not only has the potential to be utilized in the fabrication of the porous 3-D structure, but also progresses on the surface of materials because of the heterogeneous reaction between the water-insoluble PLA polymer chains and water-soluble enzyme macromolecules. Therefore, the enzymatic hydrolysis of PLA-phases proceeds through surface erosion mechanism [12].

To understand the enzymatic degradation behavior of crosslinked PLA, we investigated the enzymatic degradation of both thermoplastic (linear) and crosslinked PLAs, having pore structures with different dimensions, using proteinase-K as an effective degrading agent. Such a comparison between linear and crosslinked PLA would be worthwhile for assessing the fabrication of porous 3-D structures for tissue engineering scaffolds [10,14], because no research on the enzymatic degradation of crosslinked PLA has been reported so far.

## 2. Results and Discussion

### 2.1. Structural Characterization of Lait-X

**Figure 1:**
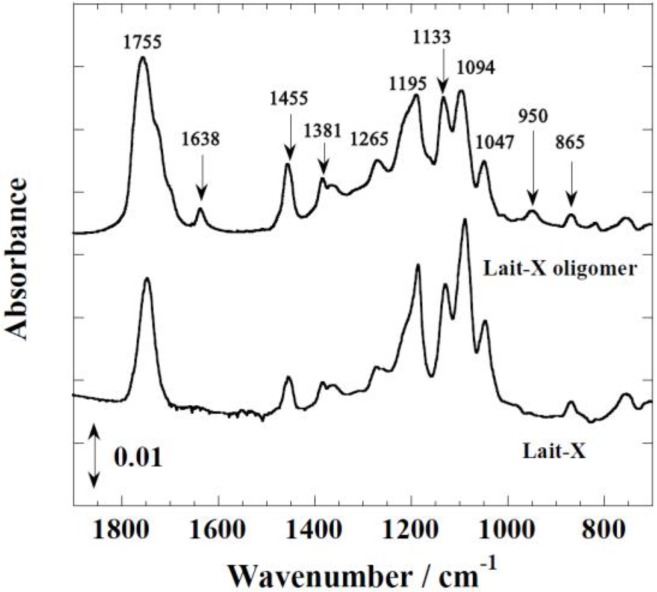
FTIR spectra of Lait-X bulk and Lait-X oligomers.

Figure 1 shows the FTIR spectra of Lait-X oligomers and Lait-X bulk after curing to confirm the formation of the crosslink network. The peaks at 1755 and 1455 cm^−1^ correspond to C=O stretching and C–H bending. The peaks at 1195, 1133, and 1094 cm^−1^ are attributed to C–O–C stretching, and C–H bending is indicated by peaks at 950 cm^−1^. The peak at 1638 cm^−1^ represents C=C stretching, which confirmed the formation of the crosslink network and the completion of curing in Lait-X oligomers.

**Figure 2:**
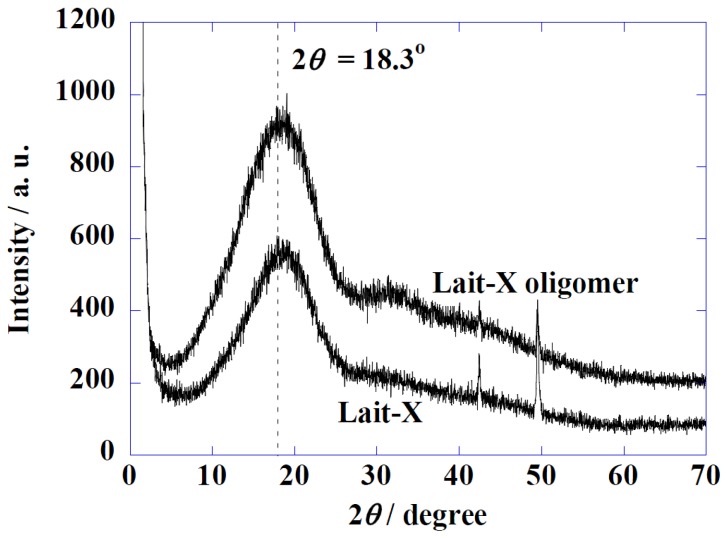
Wide-Angle X-ray Diffraction (WAXD) patterns of Lait-X oligomers and Lait-X bulk.

**Figure 3:**
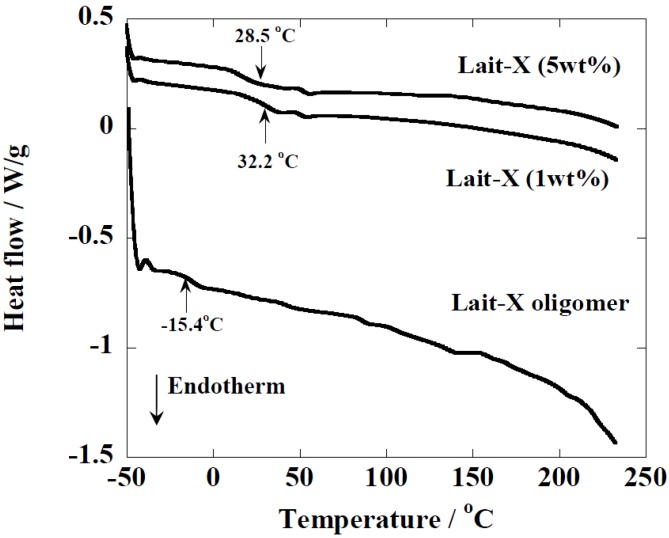
temperature-modulated DSC (TMDSC) scans of Lait-X oligomers and Lait-X bulk with different concentrations of initiator. For cured Lait-X, the content of the initiator is shown.

Figure 2 shows the Wide-Angle X-ray Diffraction (WAXD) profiles of Lait-X oligomers and Lait-X bulk (without pore structure) to analyze the crystal structures. Both samples had a shallow broad peak at 2θ = 18.3° with a correlation length of about 0.49 nm. Both samples were determined to be amorphous in nature with Lait-X having a crosslinked structure. The thermal behavior of Lait-X bulk was investigated by temperature-modulated DSC (TMDSC) (Figure 3).

The Lait-X oligomers had a *T*_g_ of −15.4 °C and the Lait-X bulk had a *T*_g_ of 32.2 and 28.5 °C when 1 and 5 wt % was used for curing. There are no melting points (*T*_m_) in the thermograms, which demonstrates that the Lait-X bulk samples were amorphous and thermoset. Also, as the initiator concentration was increased from 1 to 5 wt %, the *T*_g_ showed a decrease. The initiator to resin ratio of 1 wt % was used throughout the subsequent enzymatic degradation studies on the porous structures based on Lait-X. The *T*_g_ of the linear bulk PLA sample, designated amorphous linear PLA (χ_c_ = 0%), was 58 °C. Additionally, the density of Lait-X bulk was 1.246 g/cm^3^ and for linear PLA bulk it was 1.262 g/cm^3^ (Table 1).

**Table 1:** Morphological properties of linear and crosslinked polylactide (PLA) porous structures.

Parameters	Linear PLA Nanoporous	Linear PLA Microporous	Lait-X Porous
ρ/g·cm^−3^	1.012	0.325	0.841
average diameter/µm	0.016	0.418	0.153
*N*_p_ × 10^−8^/cell·cm^−3^	38,300	3.81	15,462
δ/µm	0.23	1.55	0.08
bulk density/g·cm^−3^	1.262	1.262	1.246
total pore area/m^2^·g^−1^	53.7	24.5	13.4
Porosity ^(a)^/%	22.8	76.4	43.0

^(a)^ Calculated by mercury porosimetry.

### 2.2. Morphological Properties

**Figure 4:**
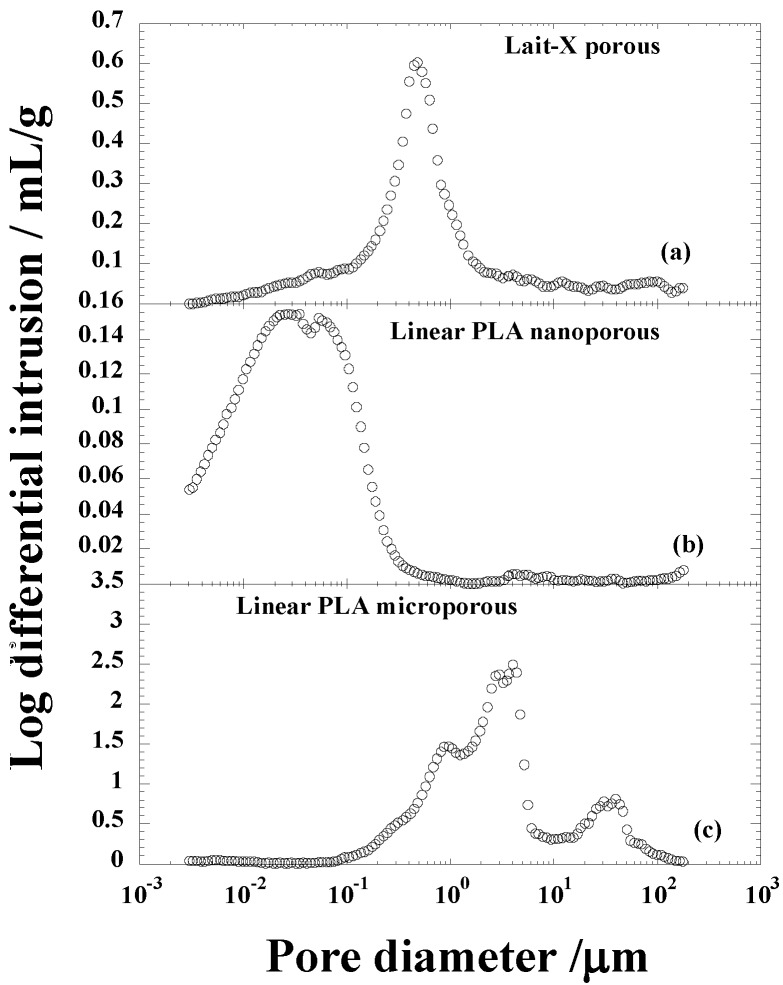
Pore size distribution of (**a**) Lait-X having a porous structure; (**b**) linear polylactide (PLA) having a nanoporous structure; and (**c**) linear PLA having a microporous structure before degradation.

The morphological parameters of the thermoset and thermoplastic PLA porous structures are summarized in Table 1. Figure 4 shows the pore size distribution of Lait-X and linear PLA samples with nanocellular and microcellular pores measured through Hg porosimetry. The pores of Lait-X were created via the salt leaching method and linear PLA via batch foam processing through supercritical CO_2_.

The average pore diameters of Lait-X porous, linear PLA nanoporous, and linear PLA microporous structures were 0.153, 0.016, and 0.418 µm, respectively; porosity (%) of 43.0, 22.8, and 76.4. The density (ρ) was 0.84, 1.01, and 0.32 g·cm^−3^, respectively. The pore density (*N*_p_) in Lait-X porous, linear PLA nanoporous, and linear PLA microporous structures were 15,462 × 10^8^, 38,300 × 10^8^, and 3.81 × 10^8^ cell·cm^−3^. The pore wall thickness of the porous walls (δ) of Lait-X porous (0.08 µm), linear PLA nanoporous (0.23 µm), and linear PLA microporous (1.55 µm) structures showed a significant variation, with Lait-X having the lowest thickness. This may be due to the difference in the preparation methods of the porous structures of Lait-X and linear PLA. The SEM images of the Lait-X porous, linear PLA microporous, and linear PLA nanoporous samples are given in Section 2.3. The Lait-X porous structure had well connected pores of varying diameters. The macroporous structures with the linear PLA had well distributed pores with thick pore walls of PLA matrix and the nanoporous ones had thin pore walls with better pore distribution than the former.

### 2.3. Enzymatic Degradation

**Figure 5:**
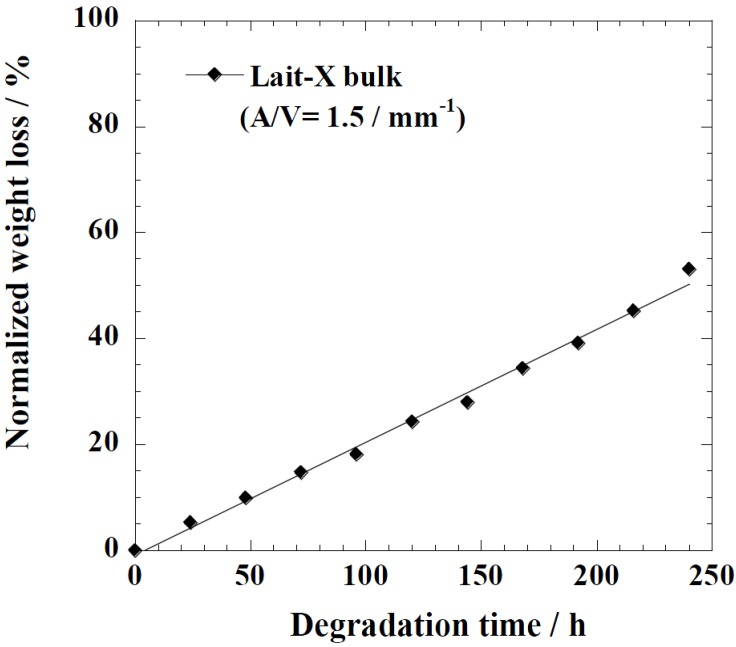
Normalized weight loss of Lait-X bulk. The solid line was calculated by linear regression.

**Figure 6:**
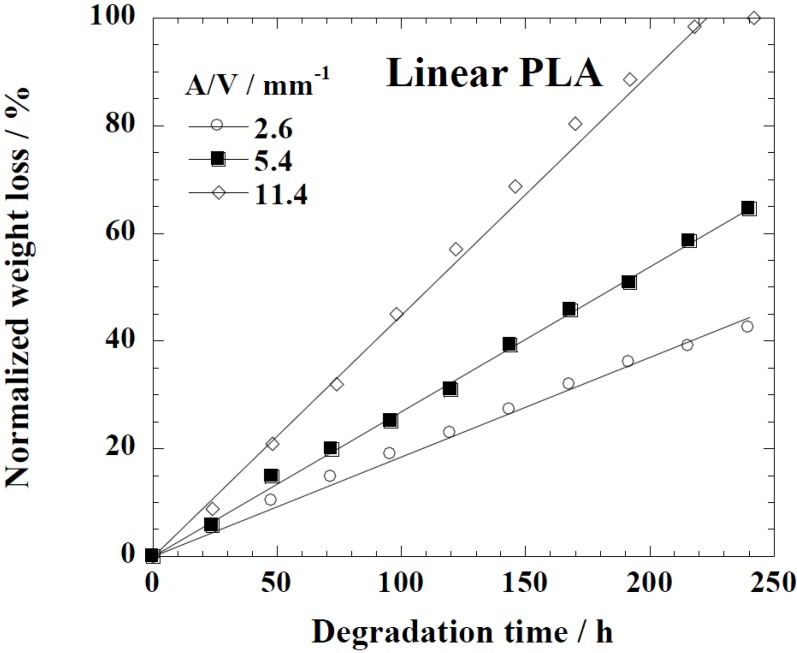
Normalized weight loss of linear polylactide (PLA) having different dimensions. The solid line was calculated by linear regression.

**Figure 7:**
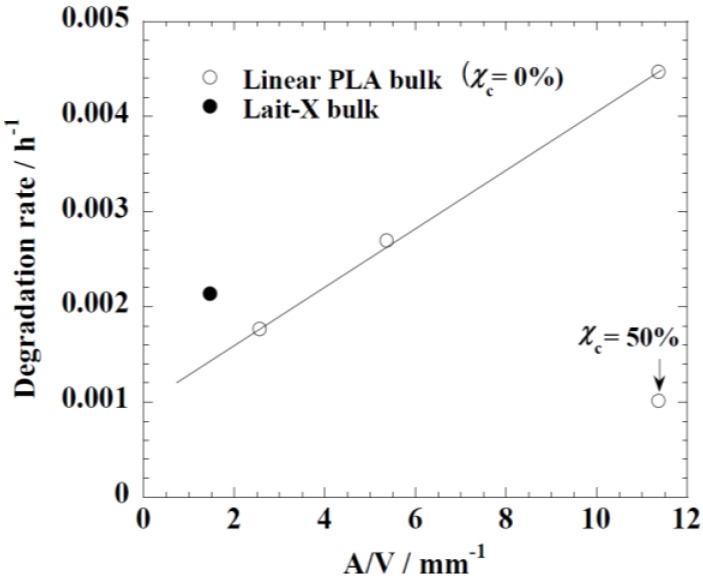
The degradation rate of linear polylactide (PLA) and Lait-X bulk. The linear PLA having χ_c_ = 50% was prepared by isothermally annealing at 100 °C for 20 min [15].

Figure 5 represents the time variation of weight loss (%) with the enzymatic degradation of the Lait-X bulk sample for 250 h. The specific surface area to volume ratio (*A*/*V*) of the sample was 1.5/mm^−1^. It is clear from the figure that weight loss increased linearly with time. This may be due to the disintegration of the crosslinked chains due to the low *T_g_* (~32 °C) that matches to the enzyme medium temperature through biodegradation. The time variation of weight loss (%) with the enzymatic degradation of the linear PLA bulk sample for different dimensions, *i.e.*, *A*/*V* ratios of 2.6, 5.4, and 11.4 mm^−1^, is give in Figure 6. When the *A*/*V* value is large (11.4 mm^−1^), the degradation is faster due to the increased interaction of the enzyme with the surface, which leads to fast degradation as compared to the lower value (2.6 mm^−1^). The degradation rate for Lait-X bulk and linear PLA samples were derived from the slopes in Figure 5 and Figure 6. The degradation rate of Lait-X bulk was faster than the linear PLA bulk with the same *A*/*V* value (Figure 7). That is, the thermoset PLA has much free volume at 37 °C, because it reaches glass transition. When the degree of crystallinity of the linear PLA was near to 50%, the degradation rate was too slow, almost 4.5 times slower than its counterpart with zero crystallinity. The linear PLA having χ_c_ = 50% was prepared by isothermal annealing at 100 °C for 20 min [15]. Thus it is clear that the enzymatic breakdown of the amorphous chains in PLA is faster than in the crystalline counterpart.

**Figure 8:**
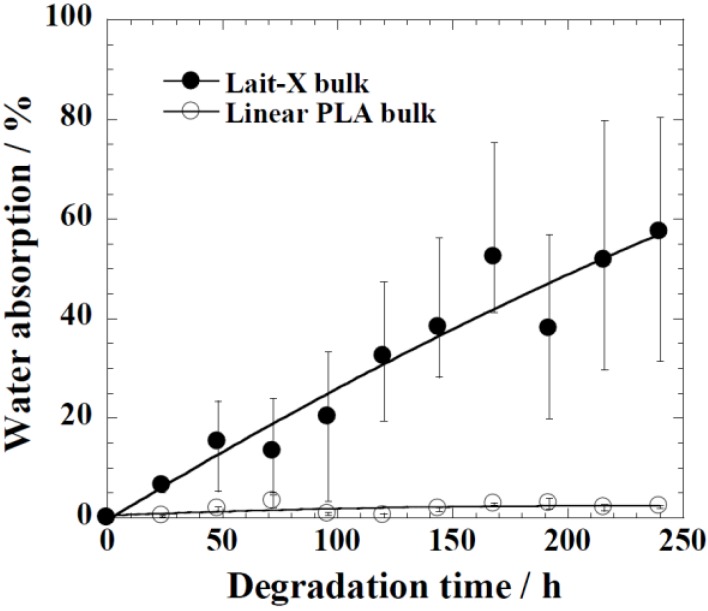
Water absorption changes of Lait-X bulk (*A*/*V* = 1.5 mm^−1^) and linear polylactide (PLA) (*A*/*V* = 2.6 mm^−1^) during the enzymatic degradation at 37 °C.

The water uptake of Lait-X bulk (*A*/*V* = 1.5 mm^−1^) and linear PLA bulk (*A*/*V* = 2.6 mm^−1^), analyzed against the degradation time, is given in Figure 8. Lait-X bulk absorbed showed nearly 60% water uptake, whereas for the linear PLA bulk, this was less than 5%. The Lait-X bulk showed significant swelling due to the crosslinked network, which led to an increased absorption. This has a significant impact on enzymatic degradation. The necessary conditions that make enzymatic reactions faster on a substrate are the temperature (37 °C) and the presence of water, which very well suites the Lait-X bulk sample.

**Figure 9:**
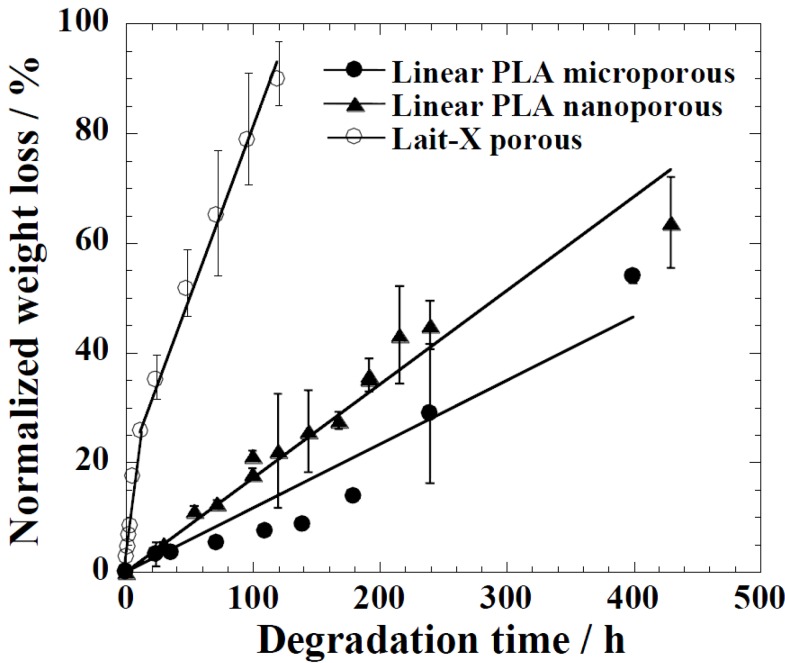
Normalized weight loss of linear polylactide (PLA) having nanoporous and microporous structures, and Lait-X having a porous structure.

The time variation of weight loss (%) with the enzymatic degradation of the Lait-X porous, linear PLA microporous, and linear PLA nanoporous samples for 250 h are shown in Figure 9. The degradation was in the order Lait-X porous > linear PLA nanoporous > linear PLA microporous with values of 2.2 × 10^−2^, 0.18 × 10^−2^, and 0.10 × 10^−2^ h^−1^, respectively, and *A*/*V* values of 1.1 × 10^4^, 5.4 × 10^4^, and 0.78 × 10^4^ mm^−1^ (Table 2). The *A*/*V* values were calculated from the parameters listed in Table 1 [15].

**Table 2:** Surface area to volume ratio (*A*/*V*) and degradation rate obtained from the initial slope in Figure 9.

Samples	*A*/V ^(a)^ × 10^−4^/mm^−1^	Degradation Rate × 10^2^/h^−1^
Lait-X porous	1.10	2.20
Linear PLA nanoporous	5.40	0.18
Linear PLA microporous	0.78	0.10

^(a)^ Calculated by using the parameters listed in Table 1 [15].

**Figure 10:**
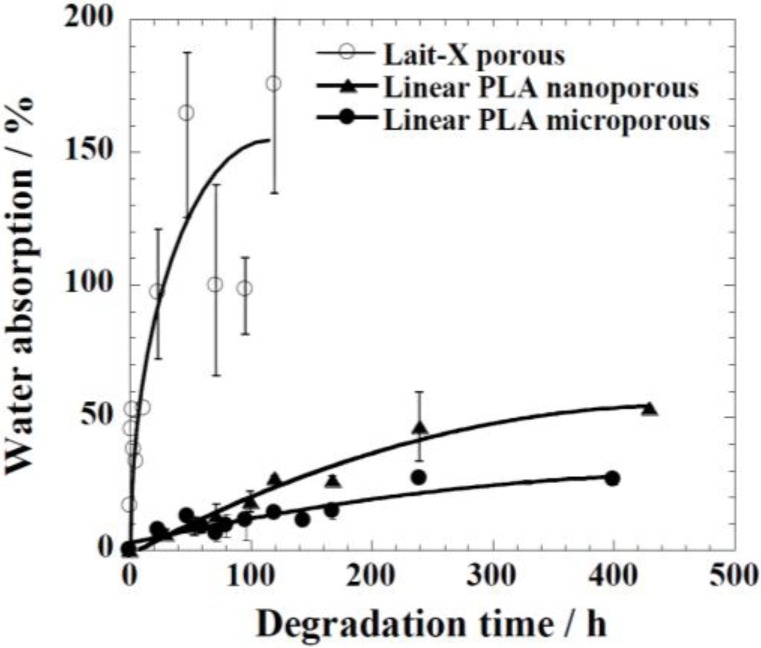
Water absorption changes of linear polylactide (PLA) having nanoporous and microporous structures, and Lait-X having a porous structure during enzymatic degradation at 37 °C.

The Lait-X porous sample showed a higher mass loss similar to bulk (Figure 9) due to the crosslinked structure with low *T_g_*. The linear PLA nanoporous sample showed a higher degradation than the microporous. This was due to a larger area of exposure for interaction with the enzyme (*A*/*V* of 5.4 × 10^−4^ *versus* 0.78 × 10^−4^ mm^−1^). The water intake of Lait-X porous, linear PLA microporous and linear PLA nanoporous samples is shown in Figure 10. The content of absorbed water greatly determines the enzymatic degradability. The water intake of the Lait-X porous structure was significantly faster than for the linear porous ones. The Lait-X porous samples reached the saturation value at around 150 h of immersion, with nearly 150% of water absorption. This is very similar to the water absorption level of hydrophilic gels. This may be attributed to the crosslinked structure, which leads to swelling in water and thus the higher water uptake. During the first 250 h, the water absorption of the linear PLA porous structures increased continuously to 30% for PLA microporous and 50% for PLA nanoporous samples. Beyond 300 h, the water uptake remained almost constant, presumably due to the saturation caused by the morphology development after enzymatic degradation. The nanoporous sample takes up larger amounts of water compared with the microporous sample. The absorbed water facilitates the enzymatic degradation of matrix linear PLA.

**Figure 11:**
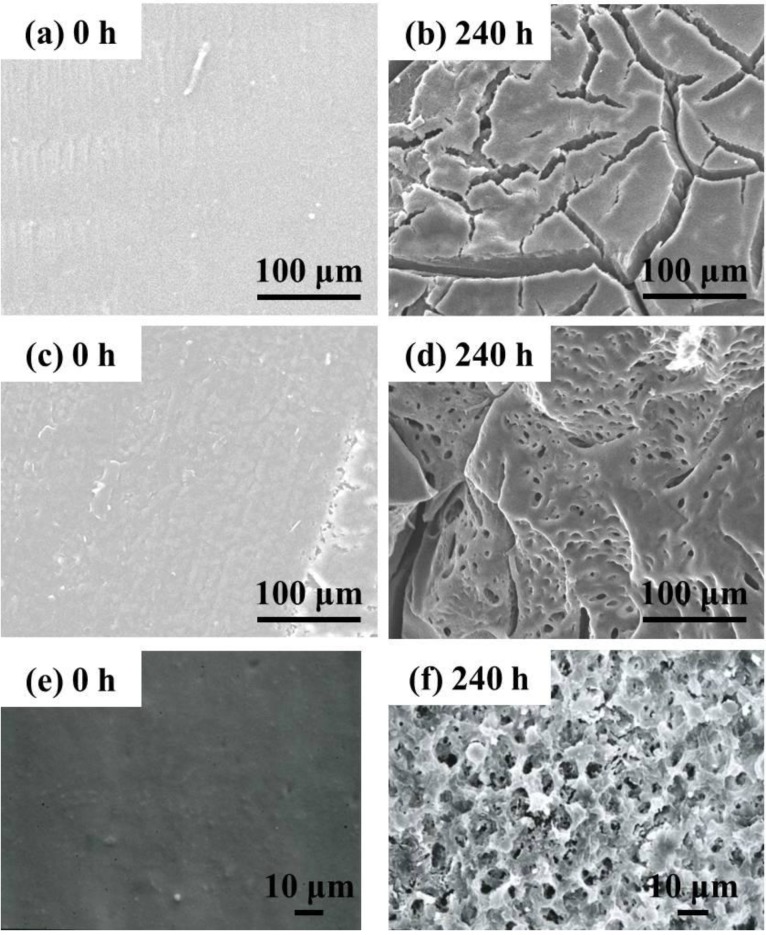
Typical SEM images of the surface and the cross section of Lait-X bulk (**a**,**b**) (surface); Lait-X bulk (**c**,**d**) (cross section); and linear polylactide (PLA) bulk (**e**,**f**) (surface) before and after enzymatic degradation for 240 h.

Figure 11 shows the surface and the cross-section morphologies of Lait-X bulk and linear PLA bulk, through SEM imaging, before and after enzymatic degradation. The surface and the cross section of Lait-X and the surface of linear PLA bulk samples are smooth before degradation (Figure 11a,c,e). After degradation up to 240 h, many pores are generated on the surface of the bulk samples and the pores are spherical in shape with circular interconnections (Figure 11d,f). This kind of connected spherical-pore structures are due to the breakdown of swollen (amorphous) regions by the enzymatic degradation, as reported previously [16,17,18].

**Figure 12:**
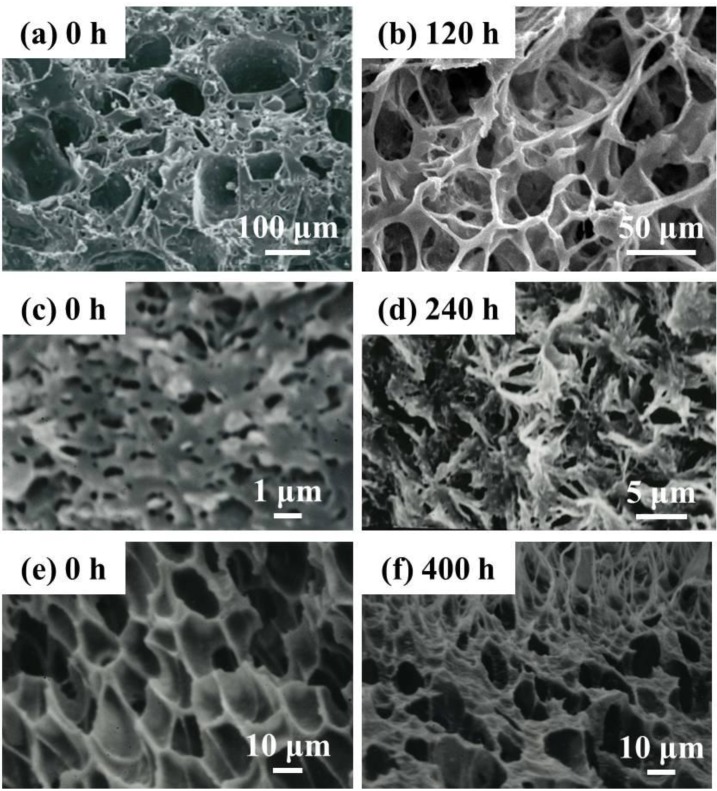
Typical SEM images of the cross section of Lait-X having porous structure (**a**,**b**), linear PLA having nanoporous structure (**c**,**d**) and linear PLA having microporous structure (**e**,**f**) before and after degradation for different time intervals.

The morphology of the Lait-X porous and linear PLA porous structures in the micro and nanoporous range is given in Figure 12. The Lait-X porous structure showed a rapid loss of features (Figure 12a,b) after 120 h of exposure to the enzymatic degradation due to the higher affinity for water that leads to enhanced activity, as discussed earlier.

Figure 12d presents the morphological change of the cross section in the PLA nanoporous sample having χ_c_ = 55% after enzymatic degradation for 240 h (corresponding to 45 wt % degradation).

For the linear PLA nanoporous structure (Figure 12d) there is a great tendency to generate the skin-layer (~50 µm thickness) with a cracked surface and hence more rapid fragmentation on the surface, as in Figure 11b. No significant change, such as pore formation, on the surface during degradation up to 240 h suggests that the core part of the foam underwent significant hydrolysis during this period. This indicates that the pore structure allows the PLA chains to become susceptible to enzymatic hydrolysis. This speculation is supported by the water uptake behavior of the PLA nanoporous sample as compared to the microporous ones. Another interesting feature of the nanoporous structure is the formation of some flower-like structures as a remaining sample in the core part, reflecting the spherulite of the crystallized PLA matrix during foaming [15].

Figure 12e shows the cross section morphologies of the PLA microporous structure with χ_c_ = 16.9% and the sample recovered after enzymatic degradation for 400 h (Figure 12f) (corresponding to 28 wt % degradation), regardless of χ_c_ in the matrix of the microporous samples. Many pores appeared on the surface after degradation for 400 h, and those with diameters of ~15 µm were shaped in a polygon cell structure. Interestingly, in the cross section, the fibrillar structures with diameters of ~1–2 µm were generated on the thick pore wall (δ ~ 1.6 µm), with some entanglement, suggesting that the amorphous region in the pore wall had been predominantly degraded. This structure was enhanced, as the degradation took place.

## 3. Materials and Methods

### 3.1. Materials

A commercial poly(l-lactide) (PLA) with a D content of 1.1%–1.7% (*M*_W_ = 150 × 10^3^ Da, *M*_W_/*M*_n_ = 1.56, *T*_g_ ≈ 60 °C, *T*_m_ ≈ 169 °C) supplied by Unitika Co., Ltd. (Osaka Japan) was dried under vacuum at room temperature. Dried pellets were converted into sheets by pressing with ~1.5 MPa at 190 °C for 3 min using a hot press. The molded sheets were quickly quenched between glass plates to prevent sheets crystallizing.

#### 3.1.1. Preparation of Crosslinked PLA Resin

**Figure 13:**
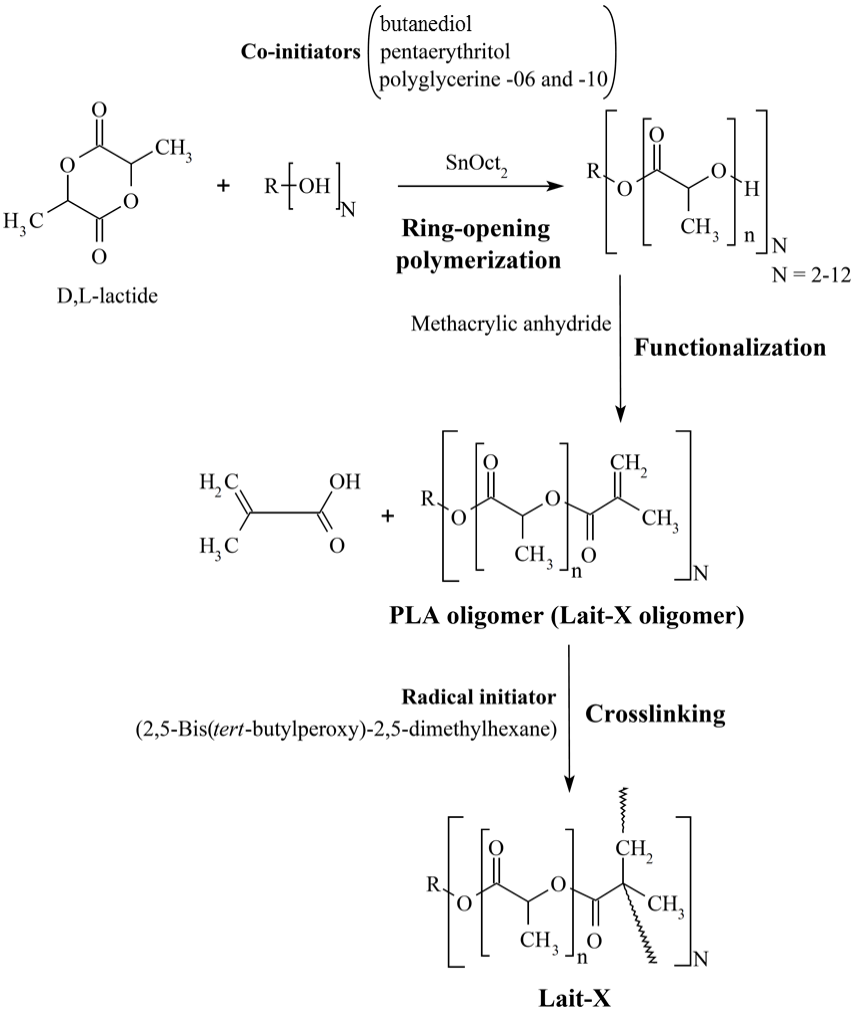
Scheme of synthesis of crosslinked polylactide (PLA).

The scheme of preparation of crosslinked PLA resin is given in Figure 13. The initial step is the synthesis of branched polylactide oligomers from PLA using a ring opening reaction with linear/branched polyols. The details of the preparation are described in our previous papers [14,19,20].

The PLA oligomers were synthesized from the polymerization of d,l-lactide in a batch reactor at 160 °C for 3 h with 0.02 mol % Sn(II) octoate as an initiator and an appropriate amount of coinitiators. The prepared telechelic d,l-lactide-based oligomers were functionalized with methacrylic anhydride for 3 h at 120 °C, and the final product was purified by distillation under reduced pressure at 140 °C. The resultant PLA oligomer was designated as Lait-X oligomer. For crosslinking, 2,5-bis(*tert*-butylperoxy)-2,5-dimethylhexane (1 wt %, Sigma-Aldrich Co., Tokyo, Japan was mixed with Lait-X resin and cured within a Teflon mold (a cubical vial of dimension: 2 × 2 × 2 cm^3^) at 150 °C for 10 h. In addition, to prevent generation of bubbles in the bulk, the sample is evacuated at 70 °C, eventually it is heated until curing temperature. The cured samples were carefully removed from the mold.

#### 3.1.2. Fabrication of Porous Structure

NaCl crystals (Salt Industry Center, Tokyo, Japan) were powdered to a particle size from 450 to 30 µm by grinding in a ceramic mortar with subsequent drying in a ceramic hot plate (CHP-170D, AS ONE Co., Tokyo, Japan) at 120 °C for one hour. The size of the NaCl particles was calculated from the polarized optical images and averaged using an image analyzing software (ImageJ; National Institutes of Health, Bethesda, MD, USA). Variation of size in the NaCl particles was preferable for generating pores with good interconnectivity between the pores. Sample suspensions prepared for the scaffold fabrication consisted of the PLA oligomer resin (Lait-X oligomer), radical initiator, and NaCl particles. The Lait-X oligomer/initiator/salt mixture (100/5/100, wt.) was stirred with a spatula for a few minutes until the mixture had completely wet the NaCl crystals. The crosslinked PLA was mixed with NaCl at around 60 °C. The resultant crosslinked PLA was designated as Lait-X. The composite mixture of resin and salt was added to a Teflon mold. A rigorous curing process was applied to all samples in a hot air oven (ST-110, ESPEC Co., Kyoto, Japan) at 150 °C for 10 h. The prepared disks were soaked in a large volume of deionized ultrapure water (specific resistance 18 MΩ·cm, TOC < 0 ppb, WR600A, YAMATO SCIENCE Co., Ltd., Kyoto, Japan) for 48 h to leach the salt particulate leaving the porous structure. The water was refreshed twice every day to favor the complete dissolution of the salt. The fabricated scaffold samples were collected, dried, and stored in a desiccator.

### 3.2. Foam Processing

Foam processing of linear PLA films was conducted in an autoclave (TSC-WC-0096, Taiatsu Tecno Co., Tokyo, Japan) by using supercritical CO_2_ [21]. PLA film (10 × 10 × 1 mm^3^ = width × length × thickness) was inserted into an autoclave (96 mL) and CO_2_ pressure was increased to 28 MPa for 4 h at 100 °C for nanocellular formation and at 120 °C for microcellular formation, respectively. For such a long time of CO_2_ dissolution into the sample, CO_2_ was already completely saturated in the sample at fixed temperature. Subsequently, the CO_2_ was quickly released from the autoclave (within 3 s). After releasing the CO_2_ pressure, the formed foam was stabilized via cooling by liquid-CO_2_ to room temperature, and then removed carefully from the autoclave and kept at ambient temperature.

### 3.3. Enzymatic Degradation

The enzymatic degradation of linear-PLA bulk, Lait-X bulk, linear-PLA foam, and Lait-X porous sample in the presence of proteinase-K was conducted according to the procedure reported by Reeve *et al.* [22]. The sheet (10 × 10 × 1 mm^3^ = width × length × thickness) was placed in a vial filed with 5 mL of 100 mM Tris-HCl buffered solution (Nacalai Tesque, Kyoto, Japan) (pH 7.9) containing 0.2 mg/mL proteinase-K (Nacalai Tesque, lyophilized powder, 36.9 units/mg) and then incubated at a thermostat-controlled temperature of 37 °C in an incubator for up to 240 h. The hydrolysis media were changed every 24 h to maintain enzymatic activity. Specimens were withdrawn at certain intervals and washed with distilled water to stop further enzymatic hydrolysis, and then dried under vacuum at room temperature for two days prior to the characterization. All measurements were performed for three replicates of specimens and averaged to obtain the final result. Moreover, crosslinked PLA bulk was also soaked in deionized ultrapure water.

The enzymatic degradation rates of these specimens were estimated by the weight loss and normalized weight loss using the following equation:

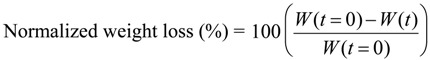
(1)
where *W* (*t* = 0), *W* (*t*) are the film weights before and after hydrolysis, respectively.

We also evaluated the water absorption according to the following equation:

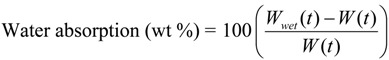
(2)


### 3.4. Characterization Methods

#### 3.4.1. Differential Scanning Calorimetry (DSC)

The specimens (10.0 mg) were characterized by using temperature-modulated DSC (TMDSC) (TA 2920; TA Instruments, New Castle, DE, USA) at the heating rate of 5 °C/min with a modulation period of 60 s and an amplitude of ±0.769 °C under nitrogen atmosphere to determine the glass transition temperature (*T*_g_), the melting temperature (*T*_m_), and heat of fusion (∆*H*). The DSC was calibrated with indium before experiments [4]. By considering the melting enthalpy of 100% crystalline thermoplastic PLA (α-phase as 93 J/g [23]), we estimated the value of the degree of crystallinity (χ_c_), calculated as ∆*H*/∆*H*_100%_ of PLA.

#### 3.4.2. Scanning Electron Microscopy (SEM)

The cell structures were investigated by using SEM (JSM-5310LV, JEOL, Tokyo, Japan). The samples were freeze-fractured in liquid nitrogen and sputter-coated with gold/platinum = 60/40 at an argon pressure of 0.1 Torr for 3 min at a current of 8 mA. The surface and cross sectional morphologies of the specimens before and after hydrolysis were also observed with SEM, operating at an accelerating voltage of 20 kV and working distance of 20 mm. The samples were sputter-coated with gold to a thickness of ~20 nm.

The pore size (2*d*), pore density (*N*_p_), and pore wall thickness (δ) were characterized from SEM images [24]. The mass density of samples was estimated by using the buoyancy method.

#### 3.4.3. Mercury Porosimetry

The pore structure and its distribution was determined by means of mercury (Hg) porosimetry (AutoPore IV-9500: Shimadzu Co., Kyoto, Japan), which covered the pore size distribution from 1 nm to 500 µm. From this measurement, porosity, total intrusion volume, total pore area, and the average pore diameters were calculated.

Washburn’s equation [25] was used to calculate the pore size distribution through mercury porosimetry as shown below:

π*r*^2^*p* = −2π*r*γ cosθ (3)

This equation assumes that the mercury intrudes a cylindrical pore. The terms *r*, γ, θ, *p* represent the radius of the cylindrical pore, surface tension, contact angle, and pressure of mercury, respectively. The contact angle (θ = 130°) and the surface tension (γ = 485 × 10^−5^ mN/m) are constants, while the radius of the cylinder (*r*) and pore size distribution are measured from the applied pressure (*p*) up to 307 MPa for the mercury intrusion.

#### 3.4.4. Wide-Angle X-ray Diffraction (WAXD)

WAXD analysis was performed for the Lait-X oligomer, Lait-X sheet and linear PLA using a Mxlabo X-ray diffractometer (MAC Science Co., Osaka, Japan, 3 kW, graphite monochromator, CuKα radiation (λ_x_ = 0.154 nm), operated at 40 kV and 20 mA) at room temperature. Samples were scanned in fixed time mode with a counting time of 2 s and diffraction angle 2θ in the range of 1°–70°.

#### 3.4.5. Fourier Transform Infrared Spectrometry (FTIR)

IR spectra were recorded using an FT730 Horiba (Kyoto, Japan) rapid-scan-type FTIR spectrometer at a resolution power of 1 cm^−1^ from 4000 cm^−1^ to 400 cm^−1^ by using thin films for crosslinked samples and KBr discs for oligomer.

## 4. Conclusions

Enzymatic degradation was conducted on linear and crosslinked PLA bulk samples along with their porous structures. The porous structures of the linear PLA samples were in the micro and nanoporous range, and prepared by batch foaming with supercritical CO_2_ and compared with the porous structures of crosslinked PLA created by the salt leaching method. The crosslinked PLA bulk samples showed a high degree of water absorption and fast degradation with the enzyme hydrolysis. This was due to the crosslinked network that assisted the swelling along with the low value of *T*_g_, which made the rupture of the crosslinks faster as compared to the linear PLA bulk samples.

The morphological analysis of Lait-X porous structure showed a rapid loss of physical features with 120 h of exposure to the enzymatic degradation due to the higher affinity for water, which led to enhanced enzymatic activity as compared to the linear PLA porous structures in the micro and nanoporous range. The water uptake behavior of the nanoporous sample was higher as compared to the microporous ones.
